# Isolation, Genomic Characterization and Pathogenicity of a European-Like PRRSV-1 Strain in Newborn Piglets from Southwestern China

**DOI:** 10.3390/vetsci13040338

**Published:** 2026-03-31

**Authors:** Xu Yang, Lei Xu, Mengjia Zhou, Weixi Li, Chenxi Hui, Pingyuan He, Hao Yang, Wenqi Yin, Yuancheng Zhou

**Affiliations:** 1 Animal Genetic Breeding and Reproduction Key Laboratory of Sichuan Province, Sichuan Animal Science Academy, Chengdu 610066, China; xuyang@scsaas.cn (X.Y.); zhoumj17@scsaas.cn (M.Z.); yanghao110@scsaas.cn (H.Y.); 411651624@scsaas.cn (W.Y.); 2 College of Animal Husbandry and Veterinary Medicine, Chengdu Agricultural College, Chengdu 611130, China; 601473235@qq.com (L.X.); 1643031603@qq.com (W.L.); 3218209396@qq.com (C.H.); 3 Veterinary Biologicals Engineering and Technology Research Center of Sichuan Province, Animtech Bioengineering Co., Ltd., Chengdu 610299, China; 2.bang@163.com

**Keywords:** PRRSV-1, pathogenicity, nsp2 deletion, BJEU06-1-like

## Abstract

Porcine reproductive and respiratory syndrome virus genotype 1 (PRRSV-1), particularly the BJEU06-1-like subgroup, has shown increasing detection in China; however, the biological characteristics of newly emerging strains in southwestern regions remain insufficiently defined. Therefore, this study aimed to isolate and characterize a PRRSV-1 strain circulating in Southwestern China and to compare its biological properties and pathogenicity with those of a representative NADC30-like PRRSV-2 strain. In this study, a PRRSV-1 field strain (CDAC-SC2025) was isolated from a lung sample collected in Sichuan Province and characterized by immunofluorescence, full-genome sequencing, and maximum-likelihood phylogenetic analysis. Recombination was assessed using RDP4 and SimPlot (Baltimore, MD, USA). Pathogenicity was evaluated in newborn piglets following intranasal challenge, with monitoring of clinical signs, viremia, viral shedding, tissue viral loads, and histopathology. CDAC-SC2025 clustered within the BJEU06-1-like subgroup and showed the closest relationship to HENZMD-10 without detectable recombination. A three-amino-acid deletion (373–375 aa) was identified in nsp2. In vivo, CDAC-SC2025 induced fever, respiratory signs, growth retardation, and mortality, but the onset of death was delayed and lesion severity was lower than those caused by the NADC30-like strain DJY. Both strains exhibited predominant viral loads in the lung and tonsils, although quantitative differences were observed across tissues. Histopathology revealed moderate lesions in CDAC-SC2025-infected piglets compared with more severe multisystemic damage caused by DJY. These findings provide updated data on the biological properties of BJEU06-1-like PRRSV-1 circulating in southwestern China.

## Simple Summary

Porcine reproductive and respiratory syndrome is one of the most damaging viral diseases affecting pigs worldwide, causing breathing problems, poor growth, reproductive failure, and economic losses for farmers. In recent years, a type of this virus commonly found in Europe has been increasingly detected in China, but its disease-causing ability and biological behavior have not been fully understood. In this study, we isolated a new strain of this virus from pigs in southwestern China and examined how it behaves in cells and in piglets. We compared it with another widely circulating strain that is known to cause more severe disease. The newly identified strain caused fever, breathing difficulties, slower weight gain, and some deaths in infected piglets, but the disease was generally milder than that caused by the comparison strain. The virus mainly replicated in the lungs and immune tissues, although differences were observed in other organs. These findings improve our understanding of newly emerging virus strains in China and provide useful information for disease monitoring, risk evaluation, and the development of more effective prevention strategies in pig production.

## 1. Introduction

Porcine reproductive and respiratory syndrome (PRRS) remains one of the most economically devastating diseases affecting the global swine industry, causing reproductive failure in sows and respiratory disorders in growing pigs [1]. The etiological agent, porcine reproductive and respiratory syndrome virus (PRRSV), is an enveloped positive-sense RNA virus belonging to the family Arteriviridae, genus *Betaarterivirus* and is characterized by a high mutation rate and frequent recombination, which drive continuous viral evolution and emergence of novel variants [2,3,4].

PRRSV is divided into two distinct genotypes: PRRSV-1 (European type) and PRRSV-2 (North American type) [5]. In China, PRRSV-2 has historically dominated the epidemiological landscape, particularly the highly pathogenic PRRSV (HP-PRRSV) lineage that emerged in 2006 and later the NADC30-like and NADC34-like variants [6,7,8,9,10]. However, increasing evidence indicates that PRRSV-1 has been detected in multiple Chinese provinces over the past decade, suggesting its gradual establishment in domestic pig populations [11,12,13]. Molecular epidemiological studies have shown that PRRSV-1 circulation is no longer sporadic and that detection rates have increased in breeding and commercial farms in several major swine-producing regions [11,14].

Phylogenetically, Chinese PRRSV-1 strains cluster into several lineages, including LV-like, HKEU16-like, BJEU06-1-like, NMEU09-1-like, and Amervac-like groups [11,15]. Among these, BJEU06-1-like strains have emerged as one of the predominant subgroups in recent years [15,16]. Genomic analyses indicate that this lineage has formed relatively stable transmission chains within Chinese production systems, and its spread is likely facilitated by inter-provincial movement of breeding pigs and intensified swine trade [11,14]. The widespread co-circulation of PRRSV-1 with diverse PRRSV-2 variants raises additional concerns, as mixed infections increase the probability of recombination, a well-recognized driver of PRRSV diversification and virulence shifts [17]. Notably, recombination events between vaccine-derived and field strains of PRRSV-1 have already been reported in Europe, highlighting the epidemiological significance of genotype coexistence [16].

Despite increasing reports of PRRSV-1 detection in China, comprehensive biological characterization of field strains remains limited, particularly in southwestern regions where intensive pig production systems may facilitate viral dissemination. Previous studies have demonstrated that PRRSV-1 strains generally exhibit lower virulence compared with highly pathogenic PRRSV-2 isolates; however, pathogenic variability exists among lineages and even among strains within the same subgroup [15,18,19]. Comparative studies between PRRSV-1 and NADC30-like PRRSV-2 strains have revealed differences in replication efficiency, tissue tropism, and lesion severity, which may influence transmission dynamics and herd-level disease impact [7,8,18].

The nonstructural protein 2 (nsp2) region of PRRSV is one of the most variable genomic regions and frequently contains deletions, insertions, and recombination breakpoints [9]. In PRRSV-2, characteristic nsp2 deletions have been used as molecular markers to define major epidemic lineages, including HP-PRRSV and NADC30-like strains [7]. Although nsp2 variation is also observed in PRRSV-1, the epidemiological and biological significance of specific deletion patterns in emerging Chinese PRRSV-1 strains remains insufficiently understood.

Given the increasing detection of PRRSV-1 and its co-circulation with PRRSV-2 in China, updated regional data integrating genomic characterization and in vivo pathogenic evaluation are essential for genotype-specific risk assessment. Therefore, the aims of this study were (i) to isolate and genetically characterize a PRRSV-1 strain circulating in Southwestern China, (ii) to determine its phylogenetic position and recombination status, and (iii) to compare its replication characteristics, pathogenicity, tissue distribution, and histopathological changes with those of a representative NADC30-like PRRSV-2 strain in newborn piglets. This study was intended to provide genotype-specific evidence for understanding the biological features and potential clinical risk of emerging PRRSV-1 strains under the current co-circulation scenario in China.

## 2. Materials and Methods

### 2.1. Samples and Virus Isolation

The sample used for virus isolation was collected from a 43-day-old piglet with acute respiratory signs from a breeding farm in Southwestern China in 2025. Before sampling, the farm had experienced sow abortions and respiratory disease in weaned piglets. According to farm records, the herd was managed as a PRRSV-negative breeding farm and had not received any PRRSV vaccination in 2025, including PRRSV-1- and PRRSV-2-based vaccines. Routine vaccination against porcine circovirus (PCV2) and *Mycoplasma hyopneumoniae* was implemented on the farm. Affected pigs showed severe respiratory signs and had been treated with florfenicol and doxycycline, together with carbaspirin calcium for fever control. To exclude other common viral pathogens, the clinical sample was tested for Getah virus (GETV), porcine bocavirus (PBoV), PRRSV-1, PRRSV-2, PCV2, and porcine circovirus (PCV3), and only PRRSV-1 was detected. The collected lung tissues were immediately transported to the laboratory under cold-chain conditions and stored at −80 °C until further processing.

PRRSV-positive lung tissues confirmed by RT-PCR were homogenized in Dulbecco’s Modified Eagle Medium (DMEM) and clarified by centrifugation. The mixtures were filtered through a 0.22 μm syringe filter (Millipore, Billerica, MA, USA). The filtered mixtures were inoculated onto a monolayer of primary porcine alveolar macrophages (PAMs). After adsorption for 2 h, the cells were incubated with fresh DMEM supplemented with 5% fetal bovine serum at 37 °C in a 5% CO_2_ incubator. The inoculated cells were blindly passaged for three generations [15]. After the discovery of cytopathic effect (CPE), the virus was purified by CPE plaque assay. Finally, virus titers were determined using Reed–Muench method [20,21].

### 2.2. Identification by Reverse Transcription PCR and Complete Gene Sequencing

To evaluate the genetic stability of CDAC-SC2025 during serial passaging and to exclude potential contamination by other swine viruses, viral RNA was extracted from the 3rd (P3), 5th (P5), 10th (P10), and 15th (P15) passages propagated in PAMs. In addition, total nucleic acids were extracted for the detection of potential DNA virus contamination.

RT-PCR assays were performed to detect potential RNA virus contamination, including PRRSV-1, NADC30-like PRRSV-2, and Getah virus (GETV), using virus-specific primers (Table 1). Conventional PCR assays were conducted to screen for common porcine DNA viruses, including PCV2, PCV3, and PRV. Amplified products were analyzed by 1% agarose gel electrophoresis (Sangon Biotech, Shanghai, China).

To determine the complete genome sequence of CDAC-SC2025, eight pairs of overlapping primers covering the entire PRRSV genome were designed and synthesized (Table 2). RT-PCR products were purified and subjected to Sanger sequencing (Sangon Biotech, Shanghai, China). Sequence assembly and editing were performed using DNAMAN (version 6.0.3.93, Lynnon Biosoft, San Ramon, CA, USA). The complete genome sequence of CDAC-SC2025 has been deposited in GenBank under accession number PZ158343. The ORF5 and nsp2 regions from different passages were further amplified and sequenced to assess nucleotide and deduced amino acid variations. Sequence alignments were performed using MEGA version 7.0 (Mega Limited, Auckland, New Zealand) with the parental P3 isolate as the reference.

### 2.3. One-Step Growth Kinetics

The replication kinetics of PRRSV CDAC-SC2025 strain were determined using Reed–Muench method in PAMs cells as previously described [22].

### 2.4. Sequence Analysis

The complete genome sequence of PRRSV CDAC-SC2025 was aligned with 32 reference PRRSV strains retrieved from GenBank using the ClustalW algorithm implemented in MEGA version 7.0 [23]. Detailed information on the 32 reference strains is provided in Supplementary Table S1. Phylogenetic trees were reconstructed in MEGA version 7.0 using the maximum-likelihood (ML) method. The best-fit nucleotide substitution model was selected according to the Bayesian information criterion (BIC) implemented in the software, and branch support was evaluated by 1000 bootstrap replicates. Bootstrap values greater than 85% are shown at the corresponding nodes [24,25].

### 2.5. Pathogenicity of PRRSV CDAC-SC2025 to Newborn Piglets

Fifteen PRRSV-negative newborn piglets, confirmed free of PRRSV, PCV2, PCV3, and PCV4 infection and without prior immunization, were randomly allocated into three groups (CDAC-SC2025 group, DJY group, and mock control group), with five piglets per group. All animals were housed under identical conditions, fed with commercial liquid milk, and provided ad libitum access to water throughout the experiment.

Piglets in the CDAC-SC2025 group and DJY group were intranasally inoculated with 10^5^ TCID_50_ of PRRSV CDAC-SC2025 and PRRSV DJY, respectively. The mock control group received an equivalent volume of DMEM via the same route. The viruses used for challenge were passage 3 (P3) stocks to minimize potential cell culture adaptation effects. All piglets were monitored daily for clinical signs, including rectal temperature, respiratory symptoms, feed intake, and general behavior. Clinical scores were assigned based on previously described PRRSV challenge models [15,26], with modifications. Each parameter was scored on a scale of 0–4, and cumulative clinical scores were calculated (Supplementary Table S2). Rectal temperature, respiratory signs, behavior, and feed intake were scored on a scale of 0–4, and a cumulative clinical score was calculated. Piglets that reached predefined humane endpoints were humanely euthanized by intravenous overdose of pentobarbital sodium (40 mg/kg) [27]. At 14 dpi, all remaining animals were euthanized using the same procedure. Tissues were immediately collected for pathological examination and viral load determination.

### 2.6. Quantitative Real-Time PCR Assay

Tissue samples from all piglets were collected aseptically and homogenized in phosphate-buffered saline (PBS). Nasal, pharyngeal, and rectal swabs were collected at 12 h intervals and eluted in 1 mL PBS. Total RNA was extracted from tissue homogenates and swab suspensions using RNAiso Plus reagent (Takara, Dalian, China) according to the manufacturer’s instructions. RNA concentration and purity were assessed using a ScanDrop spectrophotometer (Analytik Jena AG, Jena, Germany) by measuring absorbance at 260 nm and the A260/A280 ratio. Reverse transcription was performed using the PrimeScript RT Kit (Takara, Dalian, China) following the manufacturer’s instructions.

Viral loads were quantified using in-house quantitative real-time PCR (qRT-PCR) assays established in our laboratory for PRRSV-1 and NADC30-like detection. PRRSV-1 was quantified using primers targeting the ORF6 region (forward: 5′-TGGCCCCTGCCCATCACGTAG-3′; reverse: 5′-GTGCCGTTTACTGATGTTAGT-3′), whereas NADC30-like was quantified using primers targeting the ORF1 region (forward: 5′-GGAGTTGCACTGCTTTACGGT-3′; reverse: 5′-CTGAGACATCGTGTGCAGTAG-3′). Detailed qPCR amplification systems and cycling parameters are provided in Supplementary Table S3 for PRRSV-1 and Supplementary Table S4 for NADC30-llike. Viral copy numbers were calculated based on the respective standard curves and expressed as log_10_ copies/g tissue or log_10_ copies/mL swab suspension.

### 2.7. Histopathologic Examination and Immunohistochemistry

Tissue samples were collected and immediately fixed with 4% paraformaldehyde. The samples were then dehydrated, cleared, and embedded in paraffin. Sections were prepared and stained with hematoxylin and eosin (H&E). Porcine lung, kidney, liver, spleen, and mesenteric lymph nodes fixed with 4% paraformaldehyde were sent to Wuhan Servicebio Technology Co., Ltd. (Wuhan, China) for immunohistochemical analysis to observe the proliferation of PRRSV in these tissues.

## 3. Results

### 3.1. Virus Isolation, Identification, Growth Kinetics, and Genetic Stability of CDAC-SC2025

**Figure 1:**
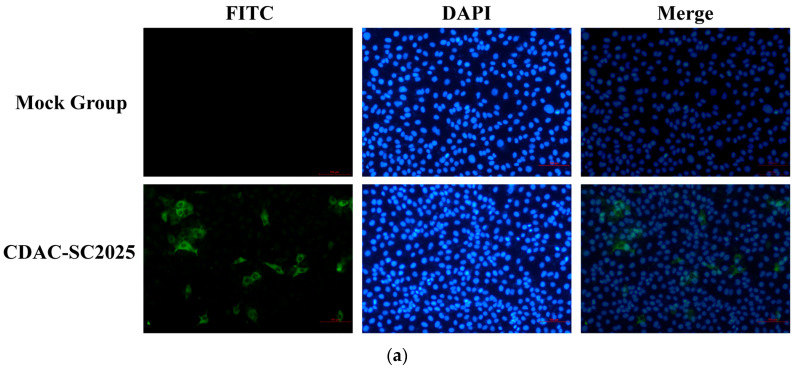

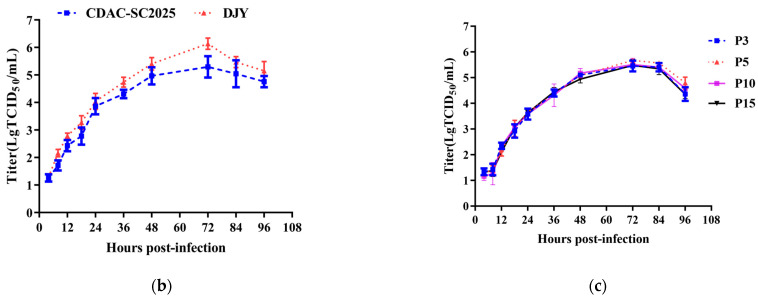
Identification and replication kinetics of CDAC-SC2025 in PAMs cells. (**a**) Indirect immunofluorescence assay (IFA) of PAMs cells infected with CDAC-SC2025. Cells were stained with anti-PRRSV-1 GP5 antibody (FITC, green) and counterstained with DAPI (blue). Mock-infected cells served as negative controls; (**b**) One-step growth curves of CDAC-SC2025 and DJY in PAMs cells. Viral titers were determined at the indicated time points and expressed as log_10_ TCID_50_/mL; (**c**) Growth kinetics of CDAC-SC2025 at different passages (P3, P5, P10, and P15) in PAMs cells. Viral titers were measured at the indicated time points and expressed as log_10_ TCID_50_/mL.

PRRSV-positive lung homogenates were inoculated onto PAMs cells for virus isolation. After three blind passages, a distinct cytopathic effect (CPE) characterized by cell rounding and detachment was observed at 48 h post-infection (hpi). The isolate was subjected to three rounds of plaque purification and designated CDAC-SC2025. Indirect immunofluorescence assay (IFA) using a rabbit polyclonal antibody against PRRSV-1 GP5 revealed strong and specific cytoplasmic fluorescence signals in CDAC-SC2025-infected PAMs cells (Figure 1a), confirming that the isolate belonged to the European genotype (PRRSV-1). One-step growth curve analysis demonstrated that CDAC-SC2025 exhibited replication kinetics comparable to those of the NADC30-like PRRSV-2 strain DJY (Figure 1b). The viral titer of CDAC-SC2025 increased rapidly after 24 hpi and reached a peak of 10^5.7^ TCID_50_/mL at 72 hpi. Although the replication trend was similar between the two strains, the peak titer of CDAC-SC2025 was consistently lower than that of DJY, suggesting relatively reduced replication efficiency in PAMs cells.

To evaluate genetic stability during serial passaging, the ORF5 and nsp2 regions from P3, P5, P10, and P15 were sequenced and compared. Sequence alignment revealed complete nucleotide and deduced amino acid identity across all analyzed passages (Table 3). No additional substitutions, insertions, or deletions were detected during in vitro propagation. Notably, the characteristic three-amino-acid deletion (373–375 aa) in nsp2 remained unchanged throughout serial passaging, indicating high genetic stability of CDAC-SC2025 in PAMs cells.

To further assess phenotypic stability, one-step growth curves were performed for P3, P5, P10, and P15 in PAMs cells. The replication kinetics exhibited highly consistent trends across all passages. The peak viral titers reached 10^5^.^6^, 10^5.7^, 10^5.6^, and 10^5.6^ TCID_50_/mL for P3, P5, P10, and P15, respectively, with no significant differences observed among passages (*p* > 0.05) (Figure 1c). These findings indicate that CDAC-SC2025 maintained stable replication capacity during serial in vitro propagation, without evidence of enhanced cell culture adaptation.

### 3.2. Phylogenetic Analysis of the CDAC-SC2025 Strain

**Figure 2:**
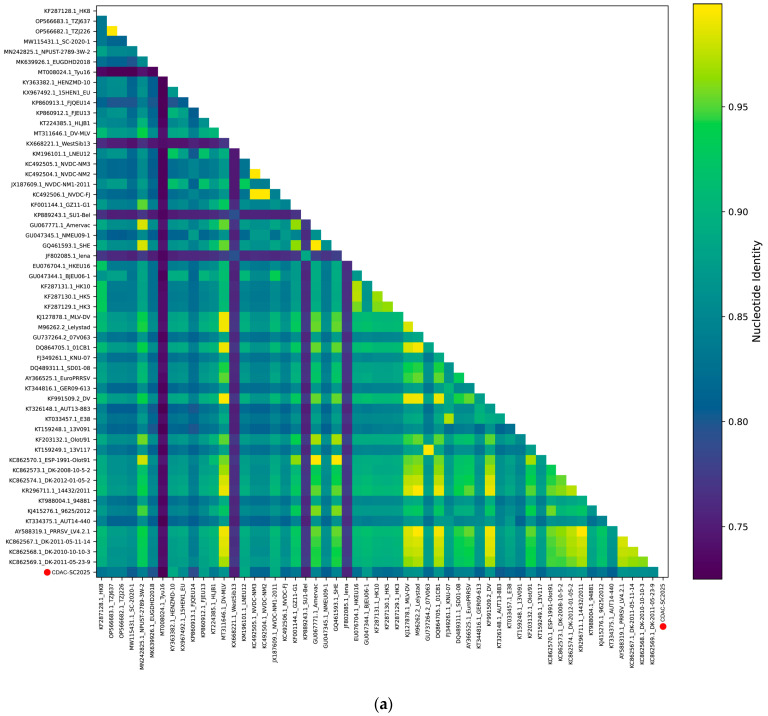

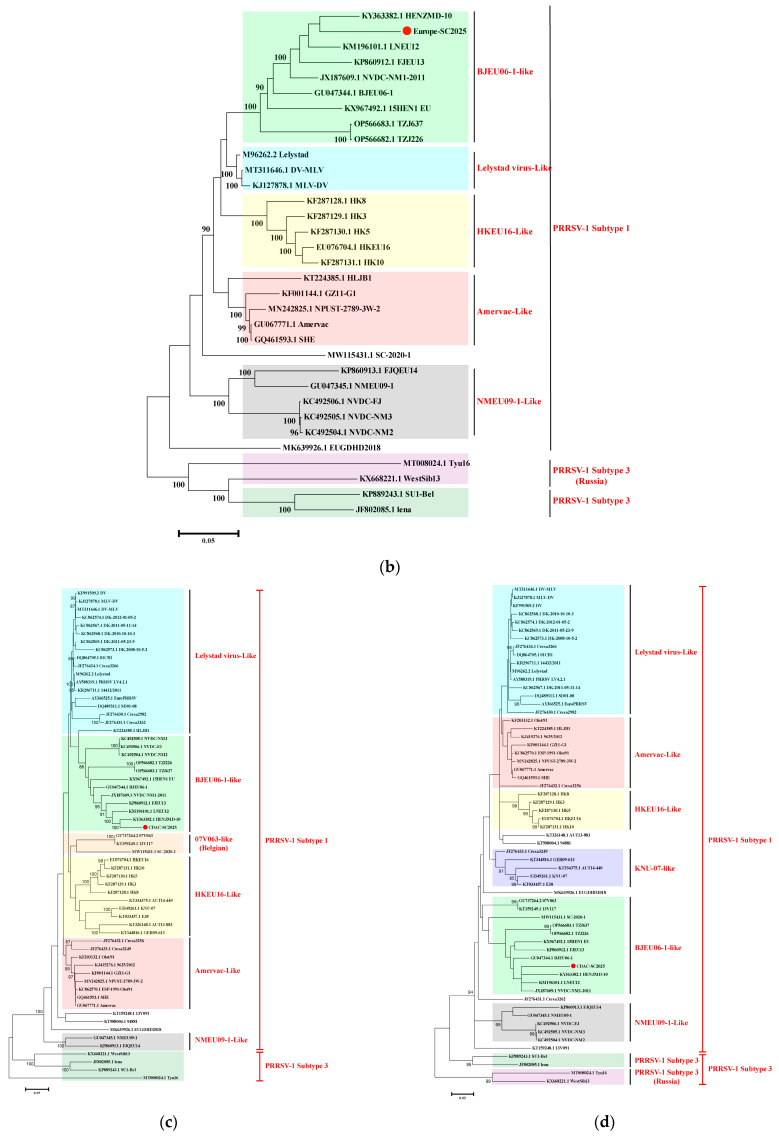

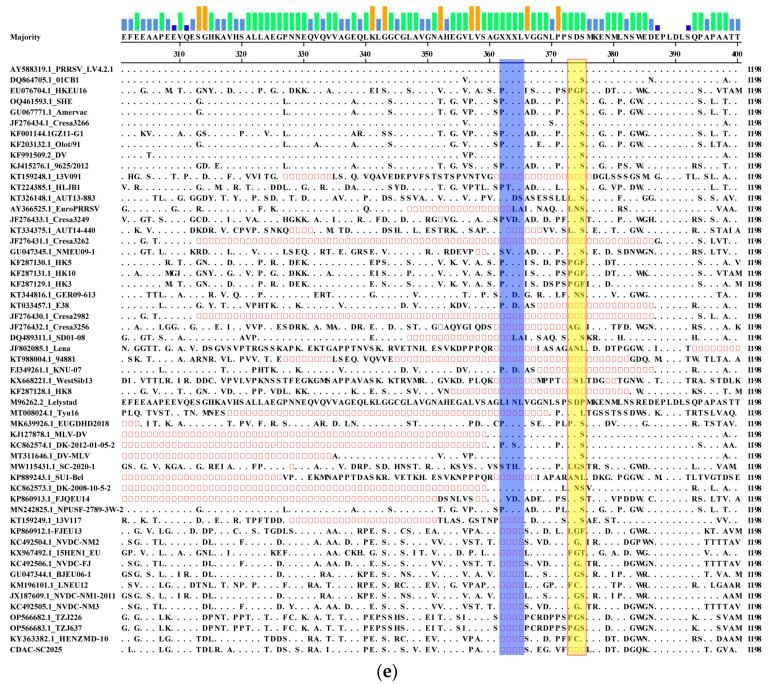
Genomic characterization and phylogenetic analysis of CDAC-SC2025. (**a**) Pairwise nucleotide identity heatmap based on complete genome sequences of CDAC-SC2025 and reference PRRSV-1 strains. (**b**) Maximum-likelihood phylogenetic tree constructed using complete genome sequences. (**c**) Maximum-likelihood phylogenetic tree based on nsp2 gene sequences. (**d**) Maximum-likelihood phylogenetic tree based on ORF5 gene sequences. (**e**) Multiple amino acid sequence alignment of the nsp2 region among CDAC-SC2025 and representative PRRSV-1 strains. The three-amino-acid deletion (positions 373–375) in CDAC-SC2025 is indicated by red boxes.

Pairwise nucleotide identity analysis based on the complete genome revealed that CDAC-SC2025 shared 72.6–89.1% nucleotide identity with the selected reference strains (Figure 2a). The highest nucleotide identity was observed with NVDC-NM1-2011 (89.1%), followed by LNEU12 (89.0%) and HENZMD-10 (89.0%), whereas the lowest identity was detected with Tyu16 (72.6%). These results indicate that CDAC-SC2025 is genetically closer to NVDC-NM1-2011, LNEU12, HENZMD-10, and other members of the BJEU06-1-like lineage, while remaining clearly divergent from several classical European prototype strains.

To further clarify its evolutionary relationship, the complete genome of CDAC-SC2025 was subjected to phylogenetic analysis. Maximum-likelihood trees were reconstructed based on the full genome, the nsp2 gene, and the ORF5 gene. All phylogenies consistently clustered CDAC-SC2025 within the BJEU06-1-like subgroup of PRRSV-1 and indicated the closest genetic relationship to the Chinese strain HENZMD-10 (Figure 2b–d).

Given that recombination events and genomic deletions are frequently reported in PRRSV, particularly within the nsp2 region, recombination analyses were performed using RDP4 and SimPlot. No statistically supported recombination events were detected by any of the seven algorithms implemented in RDP4. Sequence alignment of the nsp2 region showed that CDAC-SC2025 retained a conserved 4-amino-acid deletion shared with other representative BJEU06-1-like strains. In addition, CDAC-SC2025 harbored a characteristic 3-amino-acid deletion at positions 373–375 compared with these reference strains (Figure 2e). Together, these findings indicate that CDAC-SC2025 preserves the lineage-associated molecular signature of BJEU06-1-like PRRSV-1 strains while also possessing a distinct strain-specific deletion pattern.

### 3.3. Clinical Signs Assessment

**Figure 3:**
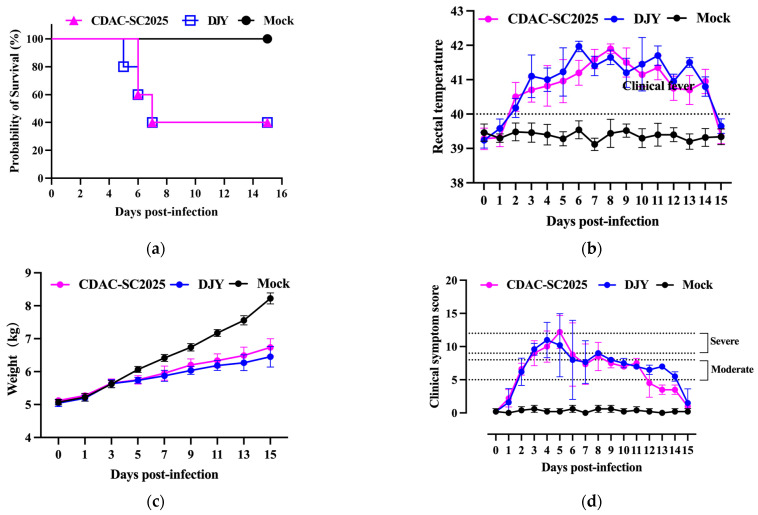
Comparative pathogenicity of CDAC-SC2025 and DJY in infected piglets. (**a**) Kaplan–Meier survival curves of piglets infected with CDAC-SC2025, DJY, or mock treatment. (**b**) Rectal temperature changes monitored daily after infection. The dotted line indicates the clinical fever threshold. (**c**) Body weight changes in piglets during the experimental period. (**d**) Daily clinical symptom scores following infection. Dotted lines indicate the predefined thresholds for moderate and severe clinical signs. Data are presented as mean ± SD.

To evaluate the in vivo pathogenicity of CDAC-SC2025, newborn piglets were experimentally infected with either the isolated PRRSV-1 strain CDAC-SC2025 or the laboratory-maintained NADC30-like PRRSV-2 strain DJY. As shown in Figure 3a, deaths in the DJY-infected group occurred earlier, beginning at 5 days post-infection (dpi), whereas deaths in the CDAC-SC2025 group were recorded starting at 6 dpi; notably, two piglets in the CDAC-SC2025 group remained alive until 15 dpi. Rectal temperature monitoring (Figure 3b) indicated that both infected groups developed fever from approximately 48 h post-infection (hpi). The DJY group reached a peak temperature of 42.1 °C at 6 dpi, whereas the CDAC-SC2025 group peaked at 42.0 °C at 8 dpi. Consistent with the febrile response, infection significantly impaired growth performance (Figure 3c). At 5 dpi, both infected groups exhibited reduced body weight gain relative to the control group, with more pronounced growth retardation observed in the DJY-infected piglets. Clinical signs were scored daily (Figure 3d). Both infected groups initially showed mild lethargy, increased respiratory rate, and occasional coughing, and the clinical manifestations progressed to marked disease by 4 dpi, characterized by severe depression, frequent coughing/wheezing, and pronounced anorexia or near-complete loss of appetite. In surviving piglets, clinical signs began to alleviate from approximately 7 dpi onward. Collectively, these results demonstrate that CDAC-SC2025 is pathogenic to newborn piglets, but it is associated with delayed onset of mortality and comparatively milder impacts on survival and growth than DJY under the conditions tested.

### 3.4. Viral Shedding Dynamics

**Figure 4:**
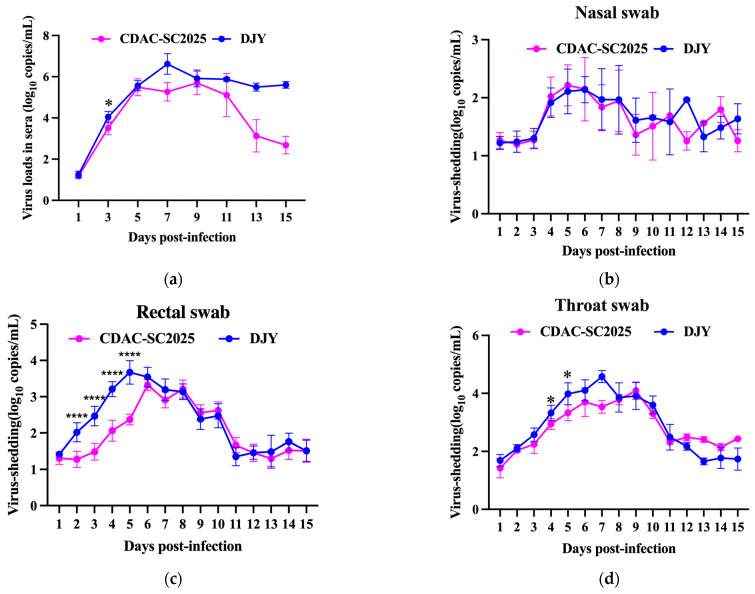
Dynamics of systemic viremia and mucosal viral shedding. (**a**) Serum viral loads. (**b**) Nasal swab viral shedding. (**c**) Rectal swab viral shedding. (**d**) Throat swab viral shedding. Viral RNA loads were determined by qRT-PCR and are expressed as log_10_ copies/mL. Data are presented as mean ± SD. Asterisks indicate statistically significant differences between the two groups at the corresponding time points. *, *p* < 0.05; ****, *p* < 0.0001.

Serum and mucosal viral loads were quantified to characterize systemic and local viral dissemination following infection with CDAC-SC2025 and DJY. As shown in Figure 4a, serum viral loads were significantly affected by both strain and time (*p* < 0.05). Viremia in the CDAC-SC2025 group peaked at 9 dpi and declined after 11 dpi. In contrast, DJY-infected piglets exhibited peak viremia earlier, at 7 dpi, and elevated viral RNA levels persisted through 15 dpi. Nasal shedding dynamics are presented in Figure 4b. Viral RNA became detectable at 4 dpi in both groups. CDAC-SC2025-infected piglets reached peak nasal shedding at 5 dpi, whereas DJY-infected piglets peaked at 6 dpi. Although both strains displayed comparable temporal shedding patterns, viral loads in the DJY group were generally higher during peak shedding. Rectal swab viral shedding results (Figure 4c) demonstrated that CDAC-SC2025 reached peak rectal viral loads at 6 dpi, whereas DJY peaked at 5 dpi. During the early and peak stages of infection, rectal viral loads in DJY-infected piglets were consistently higher than those in the CDAC-SC2025 group (*p* < 0.05). Throat swab analysis (Figure 4d) revealed detectable viral RNA shortly after infection in all challenged piglets. Peak shedding occurred at 7 dpi in the DJY group and at 9 dpi in the CDAC-SC2025 group. Statistical analysis indicated that throat viral loads in the DJY group were significantly higher during both the early and peak shedding phases (*p* < 0.05). Overall, CDAC-SC2025 exhibited a later peak of viremia and a shorter duration of elevated serum viral loads than the NADC30-like DJY strain. Systemic and mucosal viral loads were significantly lower and resolved earlier in the CDAC-SC2025 group.

### 3.5. Tissue Viral Loads and Tissue Distribution

**Figure 5:**
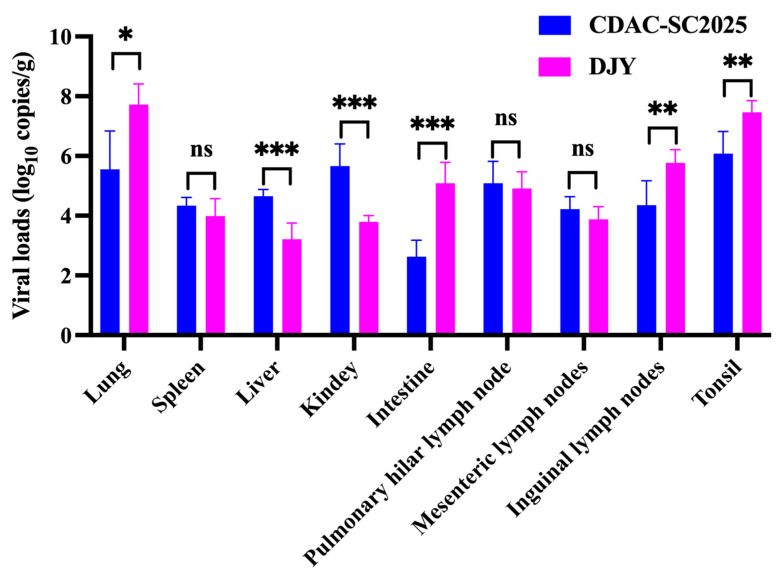
Distribution of viral RNA loads in multiple tissues of piglets. One-way ANOVA was used for analysis. *, *p* < 0.05; **, *p* <  0.01; ***, *p* <  0.001; ns, not significant.

To further characterize in vivo viral distribution, viral RNA loads were quantified in multiple organs collected at necropsy (Figure 5). Among all examined tissues, the lung exhibited the highest viral RNA levels in both groups. Lung viral loads were significantly higher in DJY-infected piglets than in those infected with CDAC-SC2025 (*p* < 0.05). Similarly, viral RNA levels in the tonsils, intestine and inguinal lymph nodes were significantly greater in the DJY group compared with the CDAC-SC2025 group (*p* < 0.05). In contrast, hepatic and renal viral RNA levels were higher in the CDAC-SC2025 group than in the DJY group (*p* < 0.05). PRRSV RNA was detectable in the spleen, pulmonary hilar lymph nodes, and mesenteric lymph nodes in piglets infected with both strains. In these tissues, viral loads were comparable between the two groups or showed no statistically significant differences. Overall, although both strains demonstrated a similar organ distribution pattern, quantitative differences in viral RNA levels were observed in respiratory, lymphoid, and certain visceral tissues.

### 3.6. Histopathological Examination

**Figure 6:**
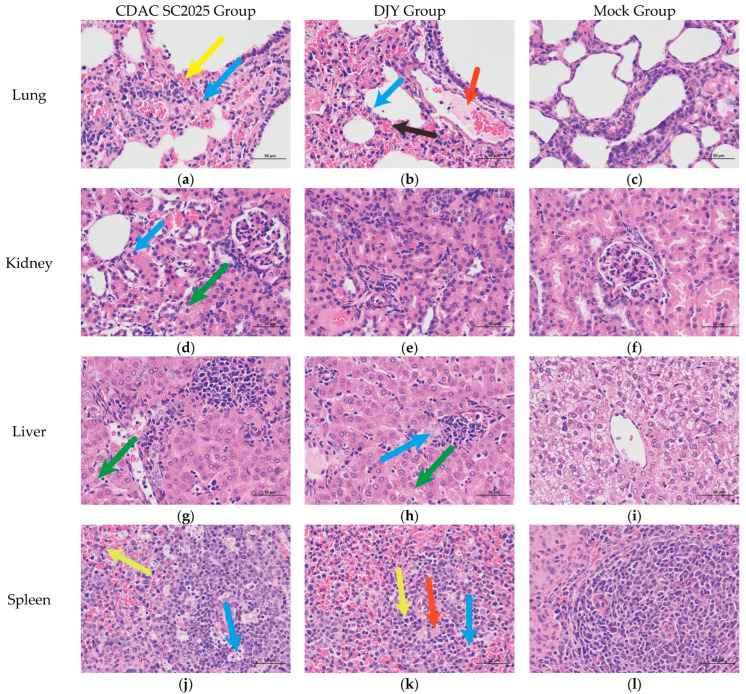

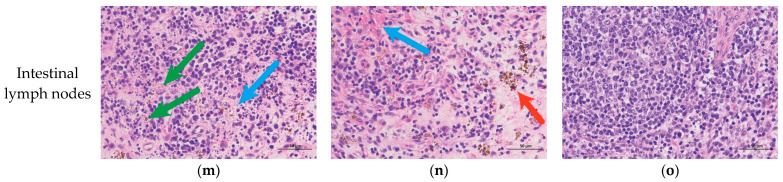
Histopathological lesions in piglets infected with PRRSV CDAC-SC2025 and DJY. Representative hematoxylin and eosin (H&E)-stained sections of lung, kidney, liver, spleen, and mesenteric lymph nodes are shown. (**a**) The lungs exhibited granulocytic infiltration (blue arrows) and capillary congestion within alveolar walls (yellow arrows) in the CDAC-SC2025 group. (**b**) The lungs showed focal inflammatory exudation (red arrows), erythrocyte extravasation (black arrows), and interstitial lymphocyte infiltration (blue arrows) in the DJY group. (**c**) Histopathological appearance of the lungs in the mock group. (**d**) The kidneys showed tubular epithelial cell swelling (green arrows) and mild vacuolar degeneration (blue arrows) in the CDAC-SC2025 group. (**e**) Renal lesions in the DJY group were characterized by tubular epithelial degeneration. (**f**) Histopathological appearance of the kidneys in the mock group. (**g**) The liver showed mild hepatocellular swelling (green arrows) in the CDAC-SC2025 group. (**h**) The liver in the DJY group exhibited hepatocellular swelling and cytoplasmic rarefaction (green arrows), as well as karyorrhexis and cytoplasmic fragmentation (blue arrows). (**i**) Histopathological appearance of the liver in the mock group. (**j**) The spleen showed scattered lymphocyte necrosis (blue arrows) and mild granulocytic infiltration (yellow arrows) in the CDAC-SC2025 group. (**k**) The spleen in the DJY group showed monocytes (blue arrows), plasma cells (yellow arrows), and fine brownish granular deposits (red arrows). (**l**) Histopathological appearance of the spleen in the mock group. (**m**) The mesenteric lymph nodes showed abundant necrotic cellular debris (blue arrows) and pigment deposition (green arrows) in the CDAC-SC2025 group. (**n**) The mesenteric lymph nodes in the DJY group showed erythrocyte infiltration (blue arrows) and yellow-brown granular aggregates (red arrows), indicative of hemosiderin deposition. (**o**) Histopathological appearance of the mesenteric lymph nodes in the mock group. Scale bars = 100 μm.

Histopathological analysis revealed distinct lesion profiles in piglets infected with CDAC-SC2025 and DJY. In CDAC-SC2025-infected piglets, the lungs exhibited mild to moderate interstitial pneumonia characterized by thickened alveolar septa, widened interstitium, mild compensatory alveolar dilatation, and focal granulocytic infiltration. Capillary congestion within the alveolar walls was evident across multiple regions, indicating localized vascular disturbance (Figure 6a). In the kidney, lesions were relatively mild, with tubular epithelial swelling, cytoplasmic rarefaction, and occasional vacuolar degeneration reflected by small intracytoplasmic vacuoles (Figure 6d). The liver displayed mild hepatocellular swelling with pale, loosened cytoplasm, accompanied by limited perivascular lymphocytic infiltration (Figure 6g). In the spleen, scattered lymphocyte necrosis with nuclear fragmentation was observed. The red pulp was uniformly distributed around the subcapsular, trabecular, and marginal regions, with a clear boundary between red and white pulp. Occasional granulocyte infiltration was noted (Figure 6j). Mesenteric lymph nodes exhibited more prominent lesions, including numerous necrotic cell debris, scattered granulocyte infiltration, and mild vascular congestion, suggesting moderate lymphoid injury (Figure 6m).

Piglets infected with the DJY strain showed more severe and widespread pathological changes compared with the CDAC-SC2025 group. In the mesenteric lymph nodes, extensive erythrocyte infiltration and abundant hemosiderin-laden deposits (yellow–brown granular aggregates) were observed, indicating hemorrhage and iron recycling due to tissue damage (Figure 6n). The lungs showed marked alveolar septal thickening, with large numbers of monocytes and lymphocytes infiltrating the interstitium, accompanied by extensive erythrocyte extravasation and focal inflammatory exudation. These findings reflect increased capillary permeability and localized hemorrhagic lesions (Figure 6b). In the spleen, scattered monocytes and plasma cells were present, along with fine brownish granules suggestive of pigment deposition or hemosiderin accumulation (Figure 6k). Renal lesions were characterized by swollen tubular epithelial cells, cytoplasmic pallor, and mild nuclear pyknosis. Some tubules showed suspected proteinaceous material retention, indicative of impaired renal filtration or tubular dysfunction (Figure 6e). The liver exhibited severe inflammatory cell infiltration, pronounced hepatocellular swelling, cytoplasmic rarefaction, and cellular disintegration, including karyorrhexis and cytoplasmic fragmentation, indicating more intense hepatocellular injury (Figure 6h).

Taken together, CDAC-SC2025 infection induced mild to moderate lesions, predominantly involving interstitial pneumonia, limited hepatocellular and renal tubular changes, and moderate lymphoid injury. In contrast, DJY induced extensive and more severe multisystemic lesions, including pronounced hemorrhage, enhanced inflammatory infiltration, and substantial tissue destruction in the lung, liver, and lymphoid organs. These findings are consistent with the higher pathogenicity observed in DJY-infected piglets compared with the European PRRSV strain CDAC-SC2025.

## 4. Discussion

In this study, we isolated and characterized a PRRSV-1 strain (CDAC-SC2025) from a swine herd in Southwestern China and evaluated its genomic features, in vitro replication kinetics, pathogenicity, and tissue distribution in newborn piglets. PRRSV continues to cause substantial economic losses worldwide. In China, PRRSV epidemiology has become increasingly complex in recent years, driven by the co-circulation of HP-PRRSV-2 and NADC30-like/NADC34-like variants, together with the expanding detection of PRRSV-1 and occasional mixed infections involving PRRSV-1 and PRRSV-2 [11,28,29,30,31,32]. BJEU06-1-like strains represent a major PRRSV-1 subgroup in China and have shown sustained circulation in key breeding regions, including Northern and Southwestern China [13]. Chinese PRRSV-1 isolates can be grouped into at least five lineages (LV-like, HKEU16-like, BJEU06-1-like, NMEU09-1-like, and Amervac-like), with BJEU06-1-like viruses frequently reported as a predominant lineage in domestic herds [13,15]. Gong et al. reported a PRRSV-1 detection rate of 14.54% in abortion-associated clinical samples collected from nine provinces [28,29]. Against this background, we characterized a newly isolated PRRSV-1 strain, CDAC-SC2025, from Southwestern China and compared its pathogenic profile with that of a representative NADC30-like PRRSV-2 strain (DJY) in newborn piglets.

Phylogenetic analyses consistently placed CDAC-SC2025 within the BJEU06-1-like lineage and showed close relatedness to strain HENZMD-10. This finding aligns with recent reports indicating that BJEU06-1-like viruses represent one of the predominant PRRSV-1 lineages in China [28,33]. Nsp2 is one of the most variable regions in the PRRSV genome and has been implicated in viral evolution, immune modulation, and virulence [3,34]. Insertions/deletions within nsp2 have been widely used as molecular signatures to differentiate PRRSV lineages, including the characteristic 1 + 29 amino acid deletion in highly pathogenic PRRSV-2, as well as the large continuous deletions seen in NADC30 and NADC34-like strains [9,11,17]. CDAC-SC2025 shared a conserved 4-aa deletion in nsp2 relative to other BJEU06-1-like reference strains. Notably, CDAC-SC2025 harbored an additional 3-aa deletion at nsp2 positions 373–375 in our alignment, distinguishing it from other BJEU06-1-like strains and representative PRRSV-1 variants. The mild-to-moderate phenotype of CDAC-SC2025 in neonatal piglets suggests that this small deletion may act as a lineage-associated molecular signature rather than a primary virulence determinant. nsp2 variation appears lineage-associated, and deletion patterns reported for BJEU06-1-like strains are distinct from those described for Amervac-like and HKEU16-like lineages. Such variation may influence virus–host interactions and immune modulation, potentially affecting persistence and transmission [13]. This unique deletion may reflect ongoing local micro-evolution in Sichuan under host immune pressure, although confirmation will require broader regional sampling and evolutionary analyses. Reverse-genetics approaches will be valuable for dissecting the functional contribution of this region within a BJEU06-1-like genetic background.

In vivo challenge experiments showed that CDAC-SC2025 is pathogenic in newborn piglets but exhibits lower overall pathogenicity than the NADC30-like PRRSV-2 strain DJY. This pattern is broadly consistent with reports that several PRRSV-1 isolates (including BJEU06-1-like and Amervac-like strains) tend to cause milder disease than many PRRSV-2 lineages (including HP-PRRSV), while still inducing respiratory signs and lung lesions in naïve pigs [15,19,35]. Lower replication in PAMs has been reported for some PRRSV-1 isolates compared with certain PRRSV-2 lineages, which could contribute to reduced pathogenicity, although this was not directly examined in the present study [14]. NADC30-like PRRSV-2 strains are often described as moderately virulent; however, they can cause substantial disease and severe lung lesions in particular host or management contexts, and pathogenicity may increase following recombination with HP-PRRSV [7,11,17].

CDAC-SC2025 produced lower levels of viremia than DJY, peaking earlier (9 dpi) and declining after 11 dpi, whereas DJY viremia peaked at 7 dpi and persisted until 15 dpi. These kinetics are consistent with reports that some BJEU06-1-like strains induce moderate viremia and tissue viral loads, while NADC30-like strains can exhibit variable replication profiles depending on their recombinant backgrounds [7,15,17]. Nasal and rectal shedding patterns observed in this study were broadly comparable to those described for other PRRSV-1 and PRRSV-2 strains in China [33]. Interestingly, throat swabs revealed a slightly delayed shedding peak for CDAC-SC2025 relative to DJY, suggesting possible differences in upper-respiratory replication or local immune responses between PRRSV-1 and PRRSV-2. Because PRRSV persistence is often linked to replication in lymphoid tissues, detectable viral loads in lymph nodes may contribute to continued shedding, although the specific anatomical drivers require further clarification [36].

Tissue viral load profiling further demonstrated strain-specific distribution patterns. Both strains exhibited the highest viral burdens in the lung, consistent with the central role of PRRSV in interstitial pneumonia and respiratory dysfunction [15,17,19]. DJY reached higher levels than CDAC-SC2025 in the lung, tonsils, and inguinal lymph nodes, indicating stronger replication within respiratory and lymphoid compartments. In contrast, CDAC-SC2025 showed relatively higher viral loads in the liver and kidney, suggesting a somewhat more pronounced visceral involvement. Similar strain-dependent variation in tissue distribution has been reported for both PRRSV-1 and PRRSV-2, with replication intensity in tonsils and lymph nodes associated with persistence and shedding potential [15,35]. These findings support the notion that strains within each genotype may display characteristic tissue tropism patterns.

Histopathology supported the replication data. CDAC-SC2025 caused mild-to-moderate interstitial pneumonia with limited hepatic and renal involvement, whereas DJY induced severe pulmonary hemorrhage, intense inflammatory infiltration, hepatocellular necrosis, and marked lymphoid damage. These findings parallel the clinical and virological differences and align with reports that NADC30-like or recombinant PRRSV-2 strains typically produce more severe systemic lesions than PRRSV-1 strains [7,17,19]. Several limitations should be acknowledged. Challenges were conducted in newborn piglets under controlled conditions and may not fully represent disease expression in older pigs or in field environments. Only one representative NADC30-like strain was included for comparison. Future studies incorporating additional strains, age groups, and reproductive models will help clarify genotype- and strain-specific pathogenicity patterns.

In conclusion, CDAC-SC2025 represents a non-recombinant BJEU06-1-like PRRSV-1 strain with a unique nsp2 3-amino-acid deletion, moderate pathogenicity in newborn piglets, and distinct tissue distribution characteristics compared with a NADC30-like PRRSV-2 strain. Under identical experimental conditions, CDAC-SC2025 showed earlier but less sustained viremia, lower viral loads in lymphoid tissues, and milder histopathological lesions, whereas DJY induced higher viral burden, prolonged shedding, and more severe multisystemic damage. This study provides direct evidence for genotype-associated differences in replication dynamics and pathogenicity between PRRSV-1 and PRRSV-2. Together with recent genomic and epidemiological data on PRRSV-1 in China [12,15,28,33], these findings highlight the clinical relevance of emerging PRRSV-1 strains, which are capable of inducing respiratory disease and tissue damage under field conditions. In particular, the co-circulation of PRRSV-1 and PRRSV-2 may increase the complexity of clinical presentation and disease management in breeding and commercial herds, supporting the need to incorporate PRRSV-1, especially BJEU06-1-like strains, into routine surveillance, molecular characterization, and vaccine evaluation programs. Further integration of reverse genetics and immunopathological studies will be essential to clarify how genomic features such as nsp2 shape virulence and immune modulation in regions where PRRSV-1 and PRRSV-2 co-circulate.

## 5. Conclusions

A PRRSV-1 strain, CDAC-SC2025, was isolated from Southwestern China and identified as a member of the BJEU06-1-like lineage without evidence of recombination. In newborn piglets, CDAC-SC2025 induced mild to moderate respiratory disease, viremia, and tissue lesions, but exhibited lower pathogenicity than the NADC30-like PRRSV-2 strain DJY. These findings indicate that emerging PRRSV-1 strains circulating in China can cause clinically relevant disease and should be considered in surveillance and control strategies under conditions of PRRSV-1 and PRRSV-2 co-circulation.

## Figures and Tables

**Table 1:** Specific primers for PCR.

Name	Primer Sequence (5′–3′)	Produce Size (bp)
PRRSV 1-F	AGAAAGCCCGGACTAACATCA	574 bp
PRRSV 1-R	ACCCCCATGTGATCGCCCTAA	
NADC30-like-F	GTGCTTGCTAGGCCGCAAGTA	175 bp
NADC30-like-R	TCTGCCACCCAACACGAGGCT	
PCV2-F	CGGATATTGTAGTCCTGGTCG	481 bp
PCV2-R	ACTGTCAAGGCTACCACAGTCA	
PCV3-F	CGACCGAGTGGGAATCTA	344 bp
PCV3-R	AGGCATCTTCTCCGCAAC	
PRV-F	AGCTGACGCTGACGACGGTCC	999 bp
PRV-R	AGACGCACACGCCCACCAGGA	
GETV-F	ACCGAAGAAGCCGAAGAAAAAGC	316 bp
GETV-R	GCACTCCAGGTCATACTTGCTC	
PRRSV-1 ORF5-F	ATGAGATGCTCTCACACATCGG	606 bp
PRRSV-1 ORF5-R	CTAGGCCTCCCATTGCTCGGC	
PRRSV-1 nsp2-1F	GCAGGAAAACGAGCTCGTGCCAAG	1631 bp
PRRSV-1 nsp2-1R	CCCCGGTCAATTAAGGCTTGT	
PRRSV-1 nsp2-2F	CGACCCTTTCGAATTTGCCGAACT	1687 bp
PRRSV-1 nsp2-2R	GAGGATAAAATGAACGAGGGTCCA	

**Table 2:** Specific primers targeting the complete gene of PRRSV-1.

Name	Primer Sequence (5′–3′)	Produce Size (bp)
PRRSV 1-1F	ATGATGTGTAGGGTATTCCCCC	1936 bp
PRRSV 1-1R	GTCCAGAATTCCTGAGGAGGTG	
PRRSV 1-2F	CCTAGCGTCTGCTTACAGACTACC	2189 bp
PRRSV 1-2R	AACGCCCCTGGGACACCACATA	
PRRSV 1-3F	CAGCGCCAACTTTGGGAACCTG	2063 bp
PRRSV 1-3R	CACAAAAGTTGAACGGTCGAGA	
PRRSV 1-4F	TTGGTTCTGGTCTTGTGACAAC	2067 bp
PRRSV 1-4R	TGGATTATTTGCTTGGATAACTC	
PRRSV 1-5F	GTGGAGGTAAAGAAATCAACTGA	2205 bp
PRRSV 1-5R	AGCCACCTTCACCATGTTTAT	
PRRSV 1-6F	GGAGGTACCAGTCCCGTCGAGG	1820 bp
PRRSV 1-6R	GGCTGTTGCCGGTCCTATACAC	
PRRSV 1-7F	AGTTGGAAGGGCTCACGTGGTC	1694 bp
PRRSV 1-7R	AGGCGAACGCCTCAGAAACC	
PRRSV 1-8F	TATTATCACCACCAAATAGACGG	2196 bp
PRRSV 1-8R	TTAATTTCGGTCACATGGTTC	

**Table 3:** Sequence comparison of ORF5 and nsp2 regions among different passages of CDAC-SC2025.

Passage	ORF5 nt Identity (%)	ORF5 aa Identity (%)	nsp2 nt Identity (%)	nsp2 aa Identity (%)	Indel Detected
P5	100	100	100	100	No
P10	100	100	100	100	No
P15	100	100	100	100	No

Note: Sequences were compared with the parental P3 isolate.

## Data Availability

Generated Statement: The original contributions presented in the study are included in the article/Supplementary Materials; further inquiries can be directed to the corresponding authors.

## Supplementary Materials

The following supporting information can be downloaded at: https://www.mdpi.com/article/10.3390/vetsci13040338/s1, Table S1: Detailed information of the reference PRRSV strains used for phylogenetic analysis; Table S2: Clinical Scoring System for PRRSV-Infected Piglets; Table S3: qPCR amplification system and rection parameters of PRRSV-1; Table S4: qPCR amplification system and rection parameters of NADC30-like.
