# Supplementary material for: Isolation, Genomic Characterization and Pathogenicity of a European-Like PRRSV-1 Strain in Newborn Piglets from Southwestern China
Source: Vet Sci. 2026 Mar 31;13(4):338. doi: 10.3390/vetsci13040338 (PMC13120401; doi:10.3390/vetsci13040338)
Supplement: Supplementary file 1 [file vetsci-13-00338-s001.zip › vetsci-4207295-supplementary.pdf]

Supplementary Table S1. Detailed information of the reference PRRSV strains used for phylogenetic analysis

GenBank accession	Strain names	Location	Isolation/Collection date
	SC2025	Sichuan, China	2025
OP566683.1	TZJ637	China	2020
OP566682.1	TZJ226	China	2020
MW115431.1	SC-2020-1	Sichuan, China	2020
MN242825.1	NPUST-2789-3W-2	Taiwan, China	2018
MK639926.1	EUGDHD2018	Guangdong, China	2018
MT008024.1	Tyu16	Russia	2016
KY363382.1	HENZMD-10	Henan, China	2016
KX967492.1	15HEN1_EU	Jiangsu, China	2015
KP860913.1	FJQEU14	Fujian, China	2015
KP860912.1	FJEU13	Fujian, China	2015
KT224385.1	HLJB1	Heilongjiang, China	2014
MT311646.1	DV-MLV	Denmark	2014
KT334375.1	AUT14-440	Austria	2014
KX668221.1	WestSib13	Russia	2013
KT326148.1	AUT13-883	Austria	2013
KT159248.1	13V091	Belgium	2013
KT159249.1	13V117	Belgium	2013
KM196101.1	LNEU12	Liaoning, China	2012
KJ415276.1	9625/2012	Hungary	2012
KC492505.1	NVDC-NM3	China	2011
KC492504.1	NVDC-NM2	China	2011
JX187609.1	NVDC-NM1-2011	China	2011
KC492506.1	NVDC-FJ	China	2011
KF001144.1	GZ11-G1	China	2011
KR296711.1	14432/2011	Hungary	2011
KC862567.1	DK-2011-05-11-14	Denmark	2011
KC862569.1	DK-2011-05-23-9	Denmark	2011
KP889243.1	SU1-Bel	Belarus	2010
KC862568.1	DK-2010-10-10-3	Denmark	2010
GU067771.1	Amervac	Spain	2009
GU047345.1	NMEU09-1	China	2009
GQ461593.1	SHE	Shanghai, China	2009
KT344816.1	GER09-613	Germany	2009
KC862573.1	DK-2008-10-5-2	Denmark	2008
KC862574.1	DK-2012-01-05-2	Denmark	2008
JF802085.1	lena	Belarus	2007
EU076704.1	HKEU16	Hong Kong, China	2007

GU737264.2	07V063	Belgium	2007
FJ349261.1	KNU-07	South Korea	2007
KT033457.1	E38	South Korea	2007
GU047344.1	BJEU06-1	Beijing, China	2006
DQ864705.1	01CB1	Thailand	2006
DQ489311.1	SD01-08	United States	2006
KT988004.1	94881	United States	2006
KF287128.1	HK8	Hong Kong, China	2004
KF287131.1	HK10	Hong Kong, China	2004
KF287130.1	HK5	Hong Kong, China	2004
AY588319.1	PRRSV_LV4.2.1	Netherlands	2004
KF287129.1	HK3	Hong Kong, China	2003
AY366525.1	EuroPRRSV	United States	2003
KJ127878.1	MLV-DV	Netherlands	1999
KF991509.2	DV	Netherlands	1996
M96262.2	Lelystad	Netherlands	1993
KF203132.1	Olot/91	Spain	1991
KC862570.1	ESP-1991-Olot91	Spain	1991

Supplementary Table S2. Clinical Scoring System for PRRSV-Infected Piglets

1. Rectal Temperature Score		2. Mental Status / Behavior Score		3. Respiratory Signs and Cough Score		4. Feed Intake Score		5. Skin and Peripheral Circulation Score	
Score	Criteria	Score	Criteria	Score	Criteria	Score	Criteria	Score	Criteria
0	Normal body temperature (≤39.5℃)	0	Alert and active	0	Normal respiration; no cough	0	Normal feed intake	0	Normal skin and mucosa
1	Mild fever (39.6-40.0℃)	1	Slight lethargy; mild depression	1	Mild tachypnea or occasional dry cough	1	Slight reduction (<25%)	1	Mild erythema or pallor
2	Moderate fever (40.1-40.5℃)	2	Marked depression, rough hair coat, reduced activity	2	Increased respiratory rate, occasional abdominal breathing, persistent mild cough	2	Moderate reduction (25%-50%)	2	Cyanosis at ear tips or extremities
3	High fever (40.6-41.0℃)	3	Severe depression, reluctant to stand, pronounced dyspnea	3	Marked dyspnea, abdominal breathing, frequent coughing/wheezing	3	Marked reduction (>50%) or refusal to eat	3	Evident cyanosis or mottled skin
4	Hyperpyrexia (>41.0℃) or fever persisting >48 h	4	Unable to stand, moribund, or dead	4	Extreme respiratory distress or near-death condition	4	Complete anorexia for >24 h or requiring supportive feeding/fluids	4	Generalized cyanosis or signs of circulatory collapse

Supplementary Table S3. qPCR amplification system and rection parameters of PRRSV-1

Reagent	Volume	Reaction parameters		
		Temperature	Time	Cycles
2×SYBR Green	10 μL	95 °C	4 min	
ddH_2O	6 μL	95 °C	15 s	35
PRRSV-F	1 μL	60 °C	15 s	
PRRSV-R	1 μL	72 °C	15 s	
cDNA	2 μL	72 °C	5 min	
Total	20 μL			

Supplementary Table S4. qPCR amplification system and rection parameters of NADC30-like

Reagent	Volume	Reaction parameters		
		Temperature	Time	Cycles
2×SYBR Green	10 μL	95 °C	4 min	
ddH_2O	6 μL	95 °C	15 s	35
PRRSV-F	1 μL	58 °C	15 s	
PRRSV-R	1 μL	72 °C	15 s	
cDNA	2 μL	72 °C	5 min	
Total	20 μL			
